# Application of DCE-MRI radiomics and heterogeneity analysis in predicting luminal and non-luminal subtypes of breast cancer

**DOI:** 10.3389/fonc.2025.1523507

**Published:** 2025-04-16

**Authors:** Ming Yao, Dingli Ye, Yuchong Wang, Tongxu Shen, Jieqiong Yan, Da Zou, Shuangyan Sun

**Affiliations:** ^1^ Department of Radiology, Jilin Cancer Hospital, Changchun, China; ^2^ Department of Radiology, The First Hospital of Jilin University, Changchun, China; ^3^ Department of Radiology, Pharmaceuticals Division, Bayer Healthcare Co. Ltd, Beijing, China

**Keywords:** radiomics, heterogeneity analysis, molecular subtype, breast cancer, magnetic resonance imaging

## Abstract

**Purpose:**

The aim of this study was to explore the application value of dynamic contrast-enhanced magnetic resonance imaging (DCE-MRI) radiomics and heterogeneity analysis in the differentiation of molecular subtypes of luminal and non-luminal breast cancer.

**Methods:**

In this retrospective study, 388 female breast cancer patients (48.37 ± 9.41 years) with luminal (n = 190) and non-luminal (n = 198) molecular subtypes who received surgical treatment at Jilin Cancer Hospital were recruited from January 2019 to June 2023. All patients underwent breast MRI scan and DCE scan before surgery. The patients were then divided into a training set (n = 272) and a validation set (n = 116) in a 7:3 ratio. The three-dimensional texture feature parameters of the breast lesion areas were extracted. Four tumor heterogeneity parameters, including type I curve proportion, type II curve proportion, type III curve proportion and tumor heterogeneity values were calculated and normalized. Five machine learning (ML) models, including the logistic regression, naive Bayes algorithm (NB), k-nearest neighbor (KNN), decision tree algorithm (DT) and extreme gradient boosting (XGBoost) model were used to process the training data and were further validated. The best ML model was selected according to the performance in the validation set.

**Results:**

In luminal subtype breast lesions, type III curve proportion and heterogeneity index were significantly lower than the corresponding parameters of the non-luminal subtype lesions both in the training set and validation set. Eight features together with four heterogeneity-related parameters with significant differences between luminal and non-luminal groups were retained as radiomics signatures for constructing the prediction model. The logistic regression ML model achieved the best performance in the validation set with the highest area under the curve value (0.93), highest accuracy (86.94%), sensitivity (87.55%) and specificity (86.25%).

**Conclusion:**

The radiomics and heterogeneity analysis based on the DCE-MRI exhibit good application value in discriminating luminal and non-luminal subtype breast cancer. The logistic regression model demonstrates the best predictive performance among various machine learning models.

## 1 Introduction

Breast cancer is the most common malignant tumor in women in China and worldwide (1). According to global cancer statistics in 2020, breast cancer accounts for 30% of female cancer cases and it is the second leading cause of cancer deaths in female (2). Breast cancer is a highly heterogeneous neoplasm, with different molecular subtypes exhibiting distinct biological behaviors. According to the St. Gallen consensus, breast cancer can be divided into luminal and non-luminal subtypes based on the expression levels of hormone receptors, which are estrogen receptor (ER), progesterone receptor (PR) and human epidermal growth factor receptor (HER2) (3). Specifically, the luminal subtype can be further subdivided into luminal A and luminal B subtypes (4).

The treatment strategies and prognosis of luminal and non-luminal breast cancer differ significantly. Therefore, the accurate differentiation of breast cancer subtypes preoperatively is important in the establishment of clinical treatment plans and prognostic evaluations. Conventionally, the immunohistochemistry (IHC) method was used to determine the molecular subtypes. However, the invasive biopsy or surgical surgery procedure is required for IHC measurements, which makes it very inconvenient, burdensome and may cause infection for breast cancer patients.

Heterogeneity analysis refers to the quantitative assessment of tissues or lesion areas in medical images to reveal the complexity and diversity of their internal structures. This study conducted a comparative analysis of dynamic enhanced breast cancer MRI images, quantitatively evaluating the contrast agent enhancement patterns within lesions at the voxel level, and calculating the heterogeneity of the lesions through a heterogeneity quantification formula. Medical image heterogeneity analysis plays a significant role in disease diagnosis, prognosis evaluation, treatment response monitoring, and research into disease pathogenesis. In the diagnosis of lung cancer, heterogeneity analysis of PET-CT images can assess the metabolic activity of tumors, providing a basis for tumor staging and prognosis evaluation. In the treatment of breast cancer, heterogeneity analysis of MRI images can monitor the tumor’s response to chemotherapy drugs and timely adjust treatment plans (5, 6). However, substantial evidence has been reported on treatment failure and disease relapse originating from intratumor heterogeneity (ITH) (7–9). Radiomics can be used to extract high-throughput quantitative imaging features that depict gray-level distribution and texture variation patterns (10, 11). To the best of our knowledge, characterization and prediction of molecular subtypes of breast cancer using combined radiomics and heterogeneity analysis have not been studied. In this study, radiomics and heterogeneity analysis is applied in dynamic contrast-enhanced magnetic resonance imaging (DCE-MRI) examinations to explore its feasibility in the prediction of molecular subtypes of luminal and non-luminal breast cancer non-invasively and quantitatively.

## 2 Materials and methods

### 2.1 Patients

**Figure 1:**
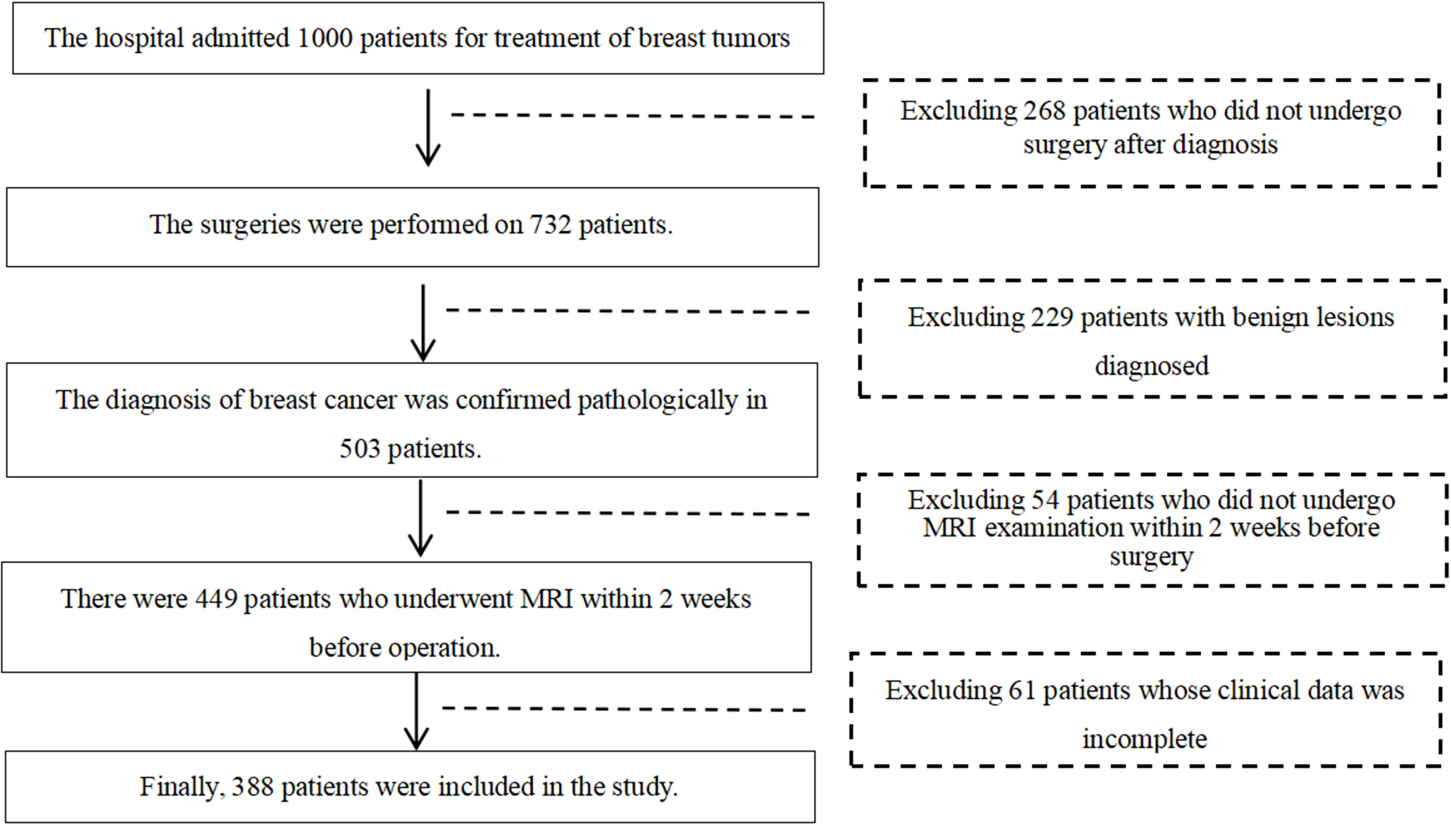
Flow chart of patient inclusion and exclusion criteria.

This retrospective study was approved by the ethics committee of Jilin Cancer Hospital, and the requirement for informed consent was waived. This retrospective study was conducted based on the pathological and imaging data of 388 female breast cancer patients who underwent surgeries at Jilin Cancer Hospital from January 2019 to June 2023. The ages of the patients were ranging from 24 to 74 years old, with a mean age of (48.37 ± 9.41) years. Most of the patients had undergone ultrasound and breast X-ray mammography examinations prior to MRI examinations. The inclusion criteria were: (1) all the recruited female patients diagnosed with breast cancer underwent surgeries; (2) all patients underwent breast MRI examination within two weeks before surgery; (3) there were pathological results that could diagnose the molecular subtypes of breast cancer. The exclusion criteria were as follows: (1) history of other malignant tumors in the body; (2) presence of significant metal artifacts or motion artifacts in MRI images. The flowchart of patient inclusion and exclusion criteria are shown in **Figure 1**.

### 2.2 MRI image acquisition

All breast MRI examinations were carried out on a Siemens Espree 1.5-T and a Philips Ingenia 3.0-T MRI scanner with a dedicated 16-channel breast MRI coil with patients in prone position. Patients’ both arms were raised with the head entering the scanner first. The intravenous bolus injection of gadolinium-based contrast agent was performed by an injector with total volume of 15 mL and a flow rate of 2.5 mL/s. It is followed by a 15 mL of saline chaser with the same flow rate.

The T1-weighted fat-suppressed sequence was performed 90 seconds after the onset of injection of contrast agent to obtain the most prominent enhancement of the tumor. The sequence was conducted with the following parameters: For the Siemens Espree 1.5-T scanner, TR = 4.65 ms, TE = 1.44 ms, flip angle = 6°, field of view (FOV) = 340 × 340 mm^2^, matrix size = 448 × 336, slice thickness = 0.9 mm, slice gap = 0.18 mm; For the Philips Ingenia 3.0-T scanner, TR = 4.6 ms, TE = 2.0 ms, flip angle = 12°, FOV = 300 × 380 mm^2^, matrix size = 300 × 380, slice thickness = 1 mm, with no slice gap.

### 2.3 Lesion segmentation, extraction of radiomics features and heterogeneity analysis

Patients were divided into two groups based on their pathological results. One group consists of luminal-type breast cancer patients, and the other group consists of non-luminal-type breast cancer patients. Since the retrospectively recruited patients were examined on two different MRI scanners, it is necessary that all acquired images were standardized and preprocessed before the feature extraction stage to eliminate the influence of inconsistent imaging parameters between two scanners. Specifically, all images were resampled using 1 mm × 1 mm × 1 mm voxels to generate standardized images with consistent slice thickness and spacing. Two physicians with more than 5 years of experience in breast MRI diagnosis analyzed the preprocessed images and determined the lesion boundaries. Both physicians were blinded to patients’ clinical characteristics.

**Figure 2:**
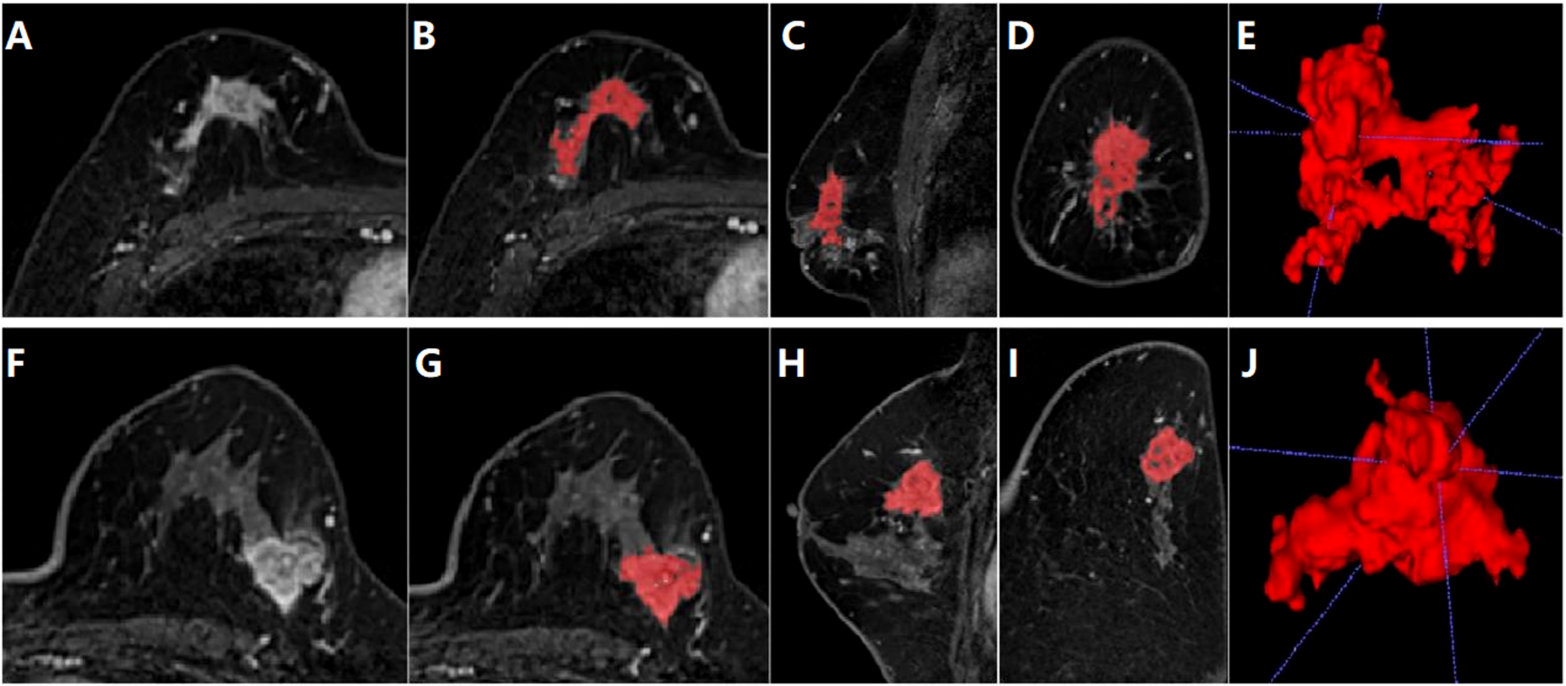
Segmentation of tumor area on DCE-MRI images of a 28-year-old female patient diagnosed with luminal type breast cancer **(A-E)** and a 52-year-old female patient diagnosed with non-luminal type breast cancer **(F-J)**. **(A, F)** The largest slice of the lesion in axial T1WI fat-suppressed images of initial 90-seconds phase. **(B, G)** Delineated and then segmented lesion ROI. **(C, H)** Segmented lesion ROI in coronal T1WI fat-suppressed images. **(D, I)** Segmented lesion ROI in sagittal T1WI fat-suppressed images. **(E, J)** 3D view of the segmented lesion ROI.

For breast tumor segmentation, the contrast-enhanced breast images with the most intense enhancement were analyzed by two radiologists with more than 5 years of experience who were blinded to the clinical outcomes to determine the lesions boundaries of each slice. The lesions region of interest (ROI) should include as much solid area as possible, while avoiding areas of vessels, cystic changes, necrosis, haemorrhage, and oedema. The ITK-SNAP software (version 3.6.0) was used to manually delineate and segment the lesions ROI along the tumor boundary of each consecutive slice covering the whole tumor (12). Thus, a three-dimensional volume of interest (VOI) of the breast tumor was obtained, as shown in **Figure 2**. For extraction of radiomics features, the AK software (Artificial Intelligence Kit, GE Healthcare, USA) was then employed to extract three-dimensional (3D) texture feature parameters from the lesion area, followed by normalization using the Min-Max scaling algorithm.

**Figure 3:**
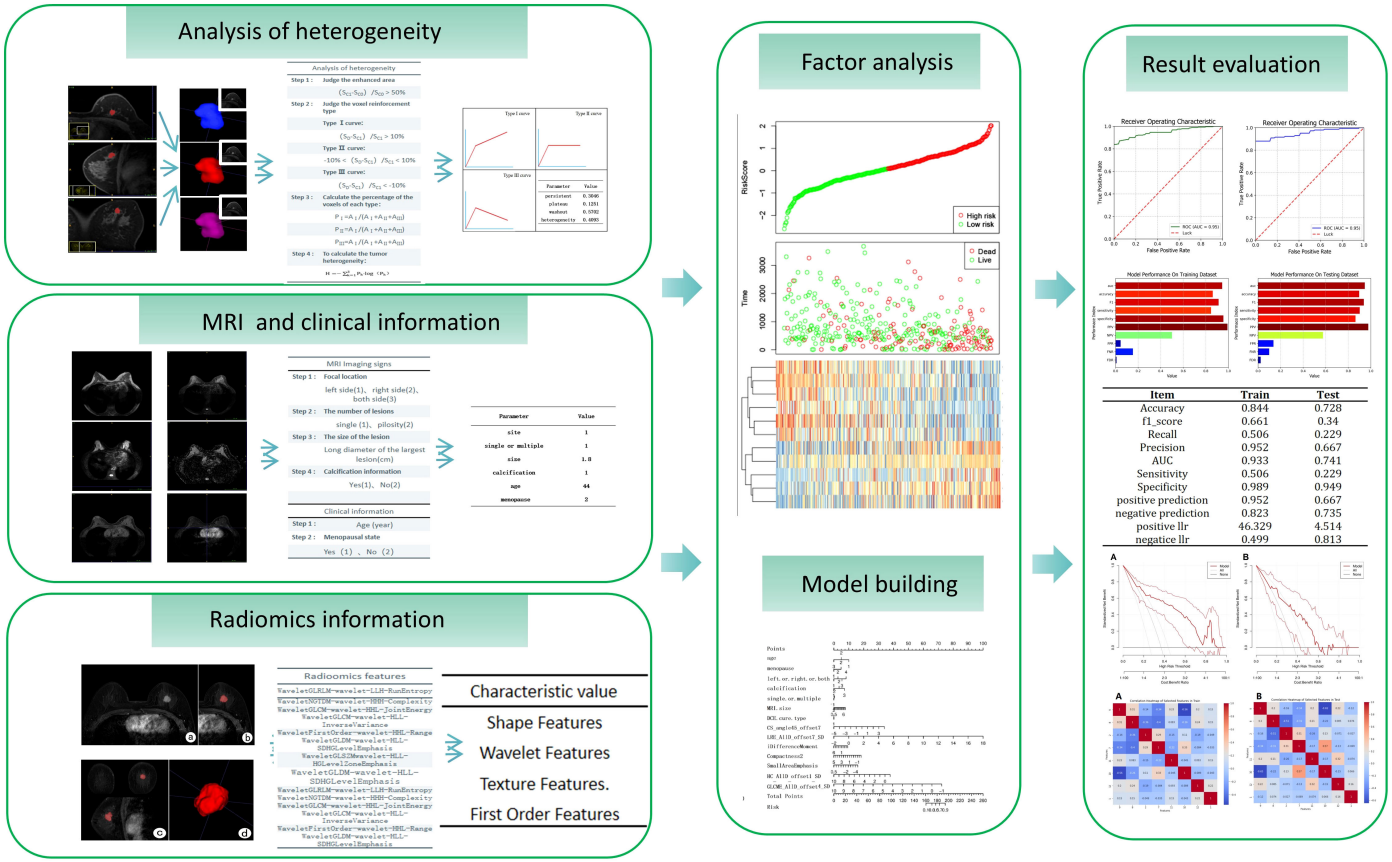
Flowchart of radiomics and heterogeneity analysis.


Dynamic enhanced MRI heterogeneity analysis utilizes image information from different time points in the lesion area to assess the heterogeneity of the lesion area. This method not only reveals the complexity of the internal structure of the lesion but also provides important information about the distribution of lesion vasculature and hemodynamics. For heterogeneity analysis, the MITK software (The Medical Imaging Interaction Toolkit) was used to calculate the signal value of each voxel in the lesion area in MRI images (13). The data from the enhanced phase, initial 90-second phase, final phase together with mask of segmented lesion ROI area were imported into the MITK software. Four heterogeneity feature parameters were calculated, which were type I curve proportion, type II curve proportion, type III curve proportion and tumor heterogeneity values. Different types of DCE-MRI curves can reflect the metabolic and heterogeneous status of the tumor. The fourth parameter is calculated by the following Equation 1. The flowchart of radiomics and heterogeneity analysis was shown in **Figure 3**.

(1) $$\text{H}=-\sum_{\text{k}=1}^{3}{\text{P}}_{\text{k}}\cdot \text{log}({\text{P}}_{\text{k}})$$

### 2.4 Radiomics feature selection and machine learning models

**Figure 4:**
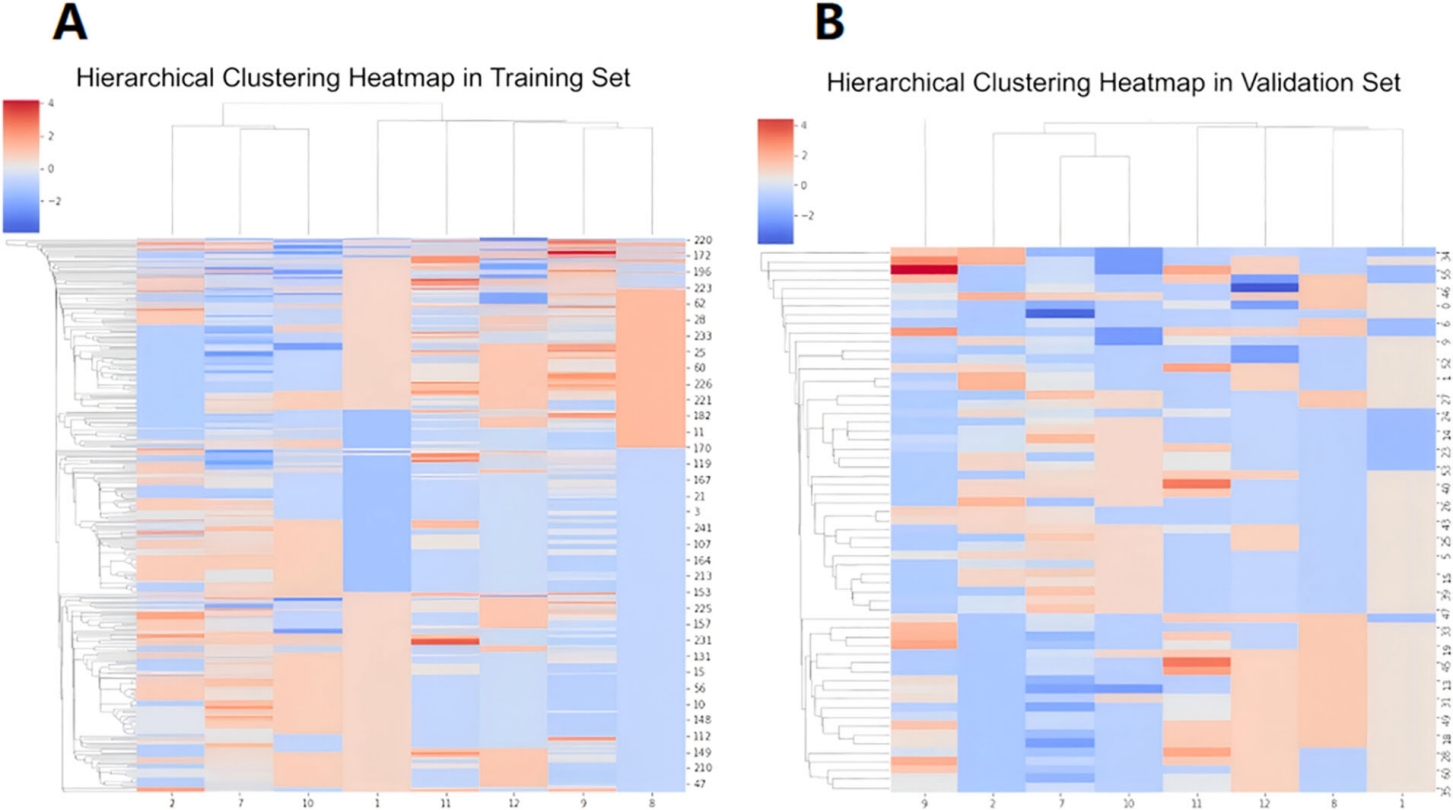
Heat maps of multivariate logistic regression analysis of machine learning model in training set **(A)** and validation set **(B)**.


The calculated radiomics feature data, heterogeneous analysis data and clinical information were standardized by Standard Scaler algorithm as depicted in Equation 2. Then, for each feature, the multiple logistic regression analysis was performed. The cutoff value was set as 0.05. The values were retained when P < 0.05.The radiomics features with significant differences in feature values were used to develop prediction models for discriminating luminal and non-luminal breast cancer. The heat maps of the multivariate logistic regression analysis in the training and validation sets were shown in **Figure 4**.

(2) $${\text{X}}^{*}=\frac{\text{X}-\text{μ}}{\text{σ}}$$

The dataset was then divided into the training set and validation set in a 7:3 ratio, which was 272 patients in the training set and 116 patients in the validation set. During the ML model training stage, five ML algorithms were employed to process the data, including the logistic regression, naive Bayes (NB), k-nearest neighbor (KNN), decision tree (DT), and extreme gradient boosting (XGBoost) models. The results of the validation set were used to evaluate the performance of the ML classifiers. The best classifier model was selected according to the accuracy of the validation set.

### 2.5 Statistical analysis

Statistical analysis was performed using SPSS software (version 25.0, IBM Corp., Armonk, NY, USA). Continuous data were expressed as mean ± standard deviation while categorical data were presented as frequencies. The differences in breast cancer molecular subtypes between two groups were compared using the *χ^2^* test. Regarding the radiomics feature consistency, inter-observer reliability was evaluated by interclass correlation coefficient (ICC) tests. The ICC is a value between 0 and 1. An ICC value below 0.5 indicates poor reliability, while value in the range 0.5-0.75 indicating moderate reliability, 0.75-0.9 demonstrating good reliability, and value above 0.9 indicating excellent reliability. Predictive performance of the machine learning models on validation sets was evaluated and compared using the area under the curve (AUC) values through receiver operating characteristic (ROC) analysis. In addition, the accuracy, sensitivity and specificity were calculated from the confusion matrices for assessments. The differences in age between the two groups were compared using student’s *t*-test. A significance level of *P* < 0.05 was considered statistically significant.

## 3 Results

A total of 388 female breast cancer patients were enrolled in this study. In the luminal breast cancer group, there were 190 patients ranging from 25 to 74 years old with an average age of 47.11 ± 8.74 years old. In the non-luminal group, there were 198 patients ranging from 24 to 74 years old with an average age of 48.96 ± 9.66 years old. The age difference between the two groups was not statistically significant (*P* = 0.074).

The enrolled patients were then divided into a training set and a validation set in a 7:3 ratio, which were 272 patients in the training set (mean age: 46.65 ± 9.64 years; range: 25-74 years) and 116 patients (mean age: 45.60 ± 8.77 years; range: 24-74 years) in the validation set. For the luminal subtype breast cancer group, type III curve proportion and heterogeneity index were significantly lower than the corresponding parameters of the non-luminal subtype group both in the training set and validation set (type III curve proportion: 0.38 ± 0.31 vs. 0.45 ± 0.32, *P = 0.033, in training set; 0.39 ± 0.18 vs. 0.46 ± 0.22, *P = 0.041, in validation set; heterogeneity index: 0.52 ± 0.26 vs. 0.65 ± 0.30, *P = 0.015, in training set; 0.53 ± 0.23 vs. 0.63 ± 0.29, *P = 0.010, in validation set). The clinical information of the patients, conventional imaging characteristics and heterogeneity analysis values of the lesions in the training and validation sets are shown in **Table 1**.

**Table 1:** Clinical information of the patients, conventional imaging characteristics and heterogeneity analysis values of the lesions.

Feature	Training set			Validation set			*P*
	Luminal group (n=133)	Non-luminal group (n=139)	*P*	Luminal group (n=57)	Non-luminal group (n=59)	*P*	
Age	45.6 ± 9.03	47.7 ± 10.25	0.043	44.3 ± 9.44	46.9 ± 8.10	0.051	0.858
Long diameter of the lesion	1.21 ± 0.65	1.40 ± 0.57	0.046	1.20 ± 0.59	1.50 ± 0.66	0.109	0.096
Number of lesions			0.861			0.788	0.806
Single shot	110 (82.7%)	105 (75.5%)		47 (82.5%)	49 (83.1%)		
Pilosity	23 (17.3%)	34 (24.5%)		10 (17.5%)	10 (16.9%)		
Calcium state			0.851			0.886	0.861
Calcification	56 (42.1%)	64 (46.0%)		22 (38.6%)	23 (39.0%)		
Without calcification	77 (57.9%)	75 (54.0%)		35 (61.4%)	36 (61.0%)		
Type I curve proportion	0.09 ± 0.09	0.07 ± 0.07	0.524	0.10 ± 0.09	0.08 ± 0.08	0.812	0.613
Type II curve proportion	0.53 ± 0.27	0.51 ± 0.28	0.591	0.52 ± 0.29	0.54 ± 0.30	0.701	0.688
Type III curve proportion	0.38 ± 0.31	0.45 ± 0.32	0.033	0.39 ± 0.18	0.46 ± 0.22	0.041	0.039
Heterogeneity quantification value	0.52 ± 0.26	0.65 ± 0.30	0.015	0.53 ± 0.23	0.63 ± 0.29	0.010	0.012

**Figure 5:**
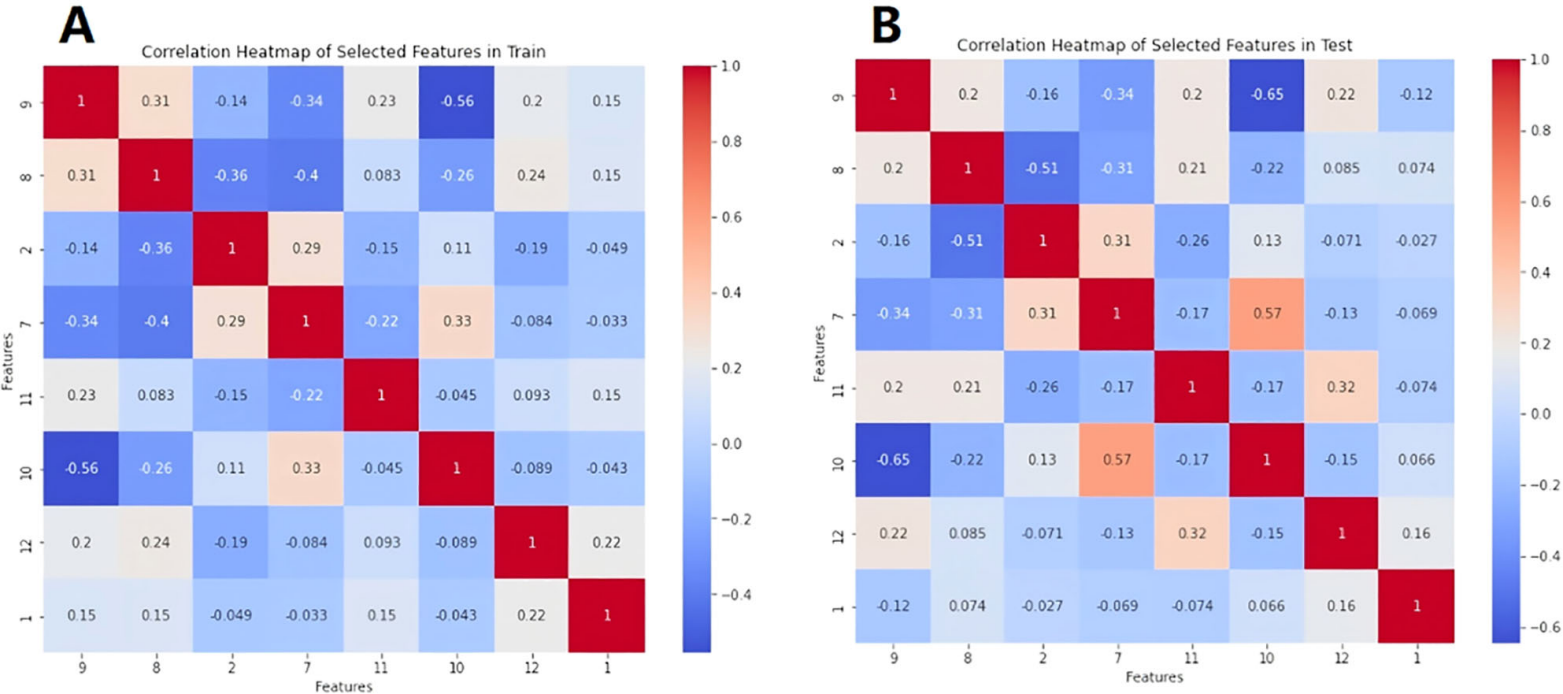
The correlation heatmaps of the 8 selected features in the training set **(A)** and validation set **(B)**.

A total of 396 texture features as well as 4 heterogeneous indices were extracted from the 3D region of the lesions. Finally, 8 features with significant differences between luminal and non-luminal breast cancer groups were retained for the prediction model, as presented in **Table 2**. For 8 texture features selected for the prediction model to differentiate luminal and non-luminal breast lesions, the ICC values of the inter-observer reliability of our research were 0.88-0.92, which suggested good consistency of radiomics features between two readers and the reliability of VOI delineation. The correlation heatmaps of the 8 selected features in the training and validation sets were shown in **Figure 5**.

**Table 2:** The eight selected features for the prediction model with significance values.

No.	Feature Name	*P*
1	HaralickCorrelation_angle0_offset1	0.0
2	Correlation_angle90_offset1	0.0
3	GLCMEntropy_AllDirection_offset4	0.0
4	ClusterShade_angle45_offset4	0.033
5	HaralickCorrelation_AllDirection_offset7_SD	0.013
6	ClusterShade_AllDirection_offset1_SD	0.007
7	Heterogeneity	0.012
8	Washout	0.039

**Figure 6:**
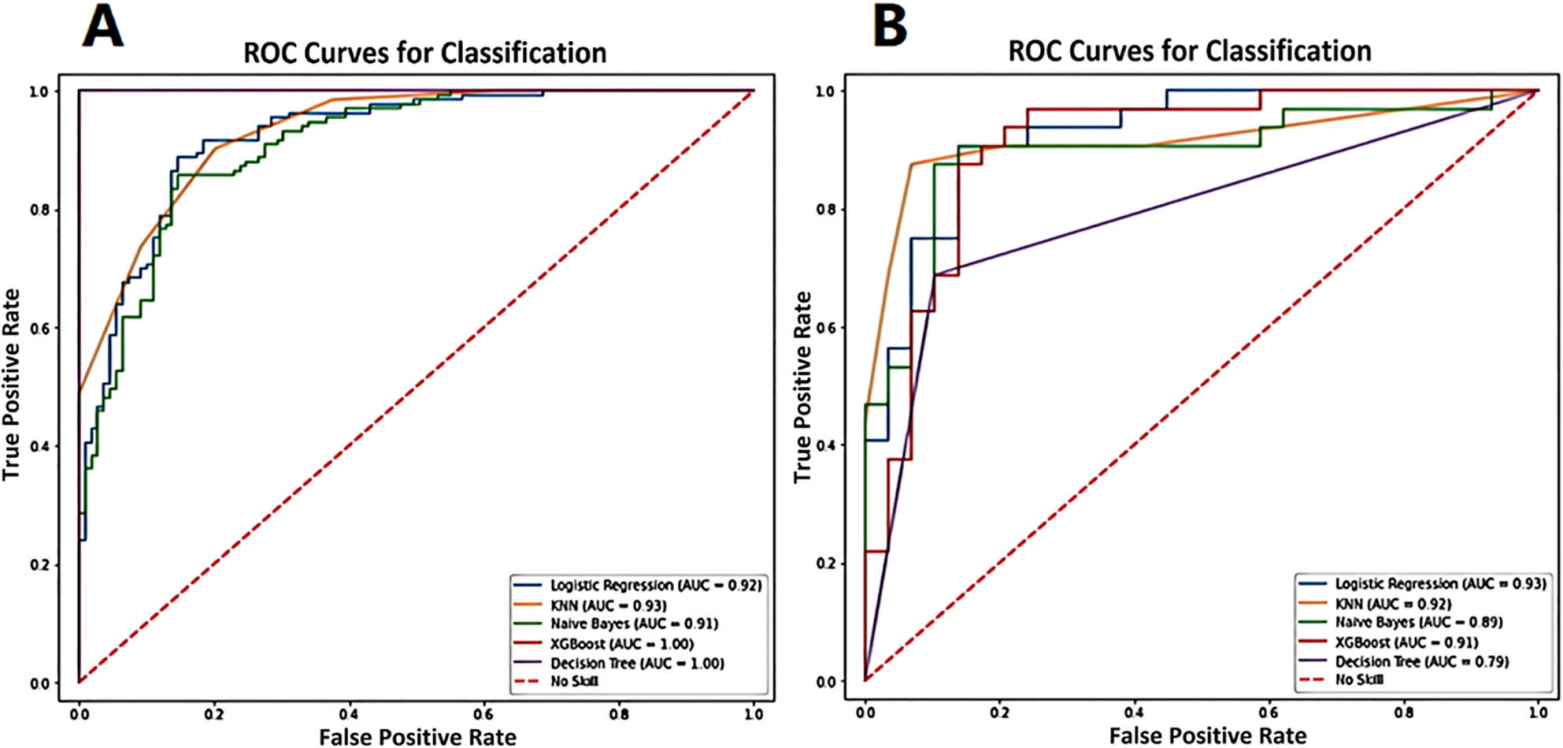
The ROC analysis of the various ML models applied in training set **(A)** and validation set **(B)** for prediction of luminal and non-luminal breast cancer.

The ROC analysis of the various ML models applied in the training and validation sets are shown in **Figure 6**. The best performance was achieved by the logistic regression model in discrimination of luminal and non-luminal breast cancer. The logistic regression ML model in the training set had the mean AUC value of 0.92 with a 95% confidence interval (CI) of 0.90 to 0.95, accuracy of 86.17%, sensitivity of 83.54% and specificity 88.73%. In the validation set, the logistic regression ML model achieved the best performance with the highest AUC value (0.93 with a 95% CI of 0.91 to 0.94), highest accuracy (86.94%), highest sensitivity (87.55%) and the highest specificity (86.25%), as shown in **Table 3**. The precision, recall and F1 score of each ML model were also calculated and shown in **Table 3**. A total of 45 breast cancer patients from Jilin Cancer Hospital were used as the external validation set. There were 25 patients with luminal type breast cancer and 20 non-luminal type breast cancer patients. The ROC analysis of the 5 machine learning models applied in the external validation set were conducted. The logistic regression ML model also achieved the best diagnostic performance with the highest AUC value of 0.88 and accuracy of 0.84. The practicability of logistic regression ML model is confirmed by external validation.

**Table 3:** The diagnostic efficacy of 5 machine learning models in the training set and validation set.

Index		AUC^*^	Accuracy	Sensitivity	Specificity	Precision	Recall	F1
Training set	DT	1 (0.99, 1.00)	0.96	0.76	0.66	0.85	0.86	0.81
	Logistic	0.92 (0.90, 0.95)	0.86	0.84	0.89	0.78	0.75	0.77
	XGBoost	1 (0.99, 1.00)	0.97	0.82	0.79	0.91	0.89	0.87
	KNN	0.93 (0.92, 0.95)	0.86	0.88	0.75	0.75	0.46	0.62
	NB	0.91 (0.90, 0.93)	0.83	0.70	0.56	0.69	0.56	0.62
Validation set	DT	0.79 (0.78, 0.81)	0.80	0.83	0.62	0.53	0.45	0.48
	Logistic	0.93 (0.91, 0.94)	0.87	0.88	0.86	0.76	0.75	0.67
	XGBoost	0.91 (0.90, 0.92)	0.80	0.87	0.79	0.58	0.58	0.58
	KNN	0.92 (0.90, 0.93)	0.83	0.86	0.71	0.75	0.38	0.52
	NB	0.89 (0.87, 0.90)	0.82	0.61	0.51	0.61	0.45	0.51

*Data in parentheses are 95% confidence intervals.

**Figure 7:**
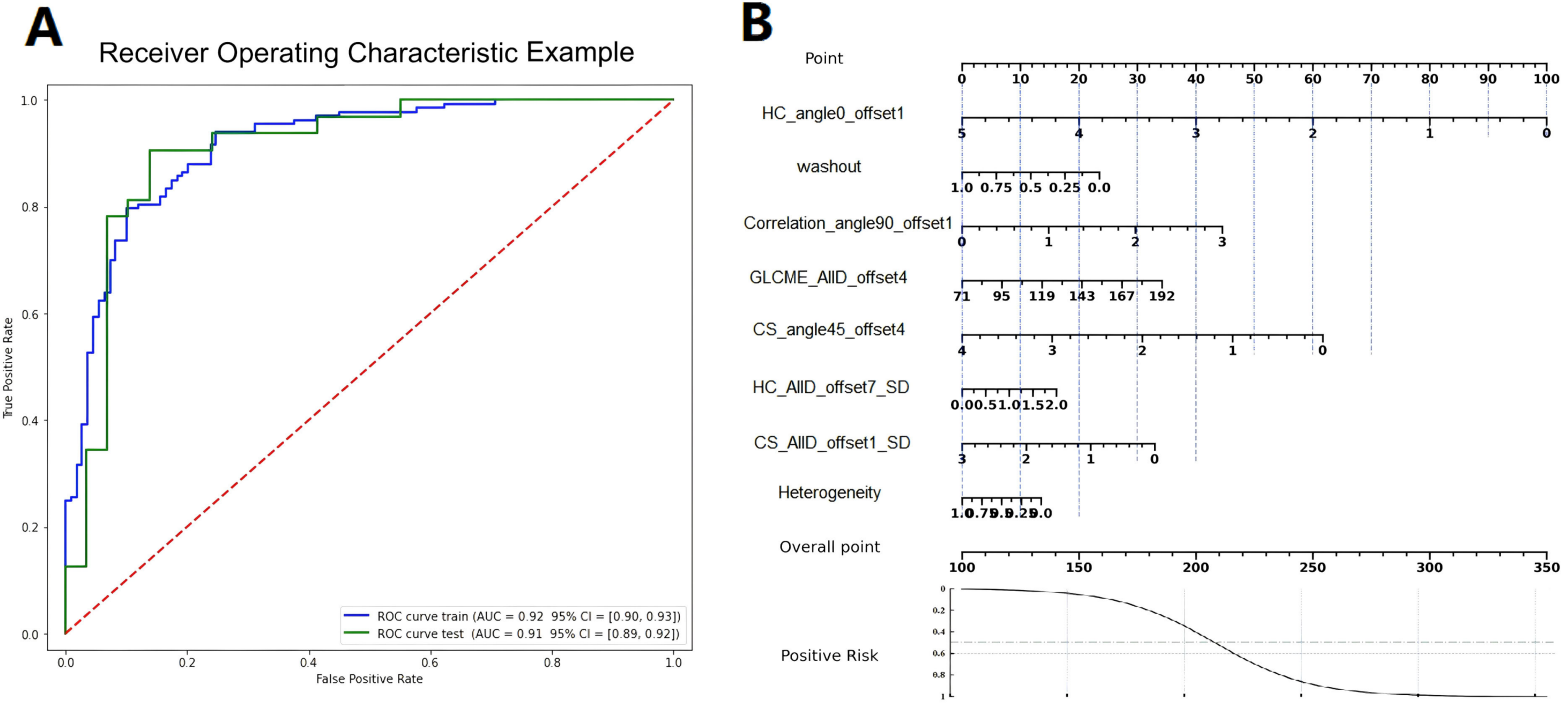
The ROC curves **(A)** and nomograms **(B)** of the logistic regression model in the training and validation sets.

**Figure 8:**
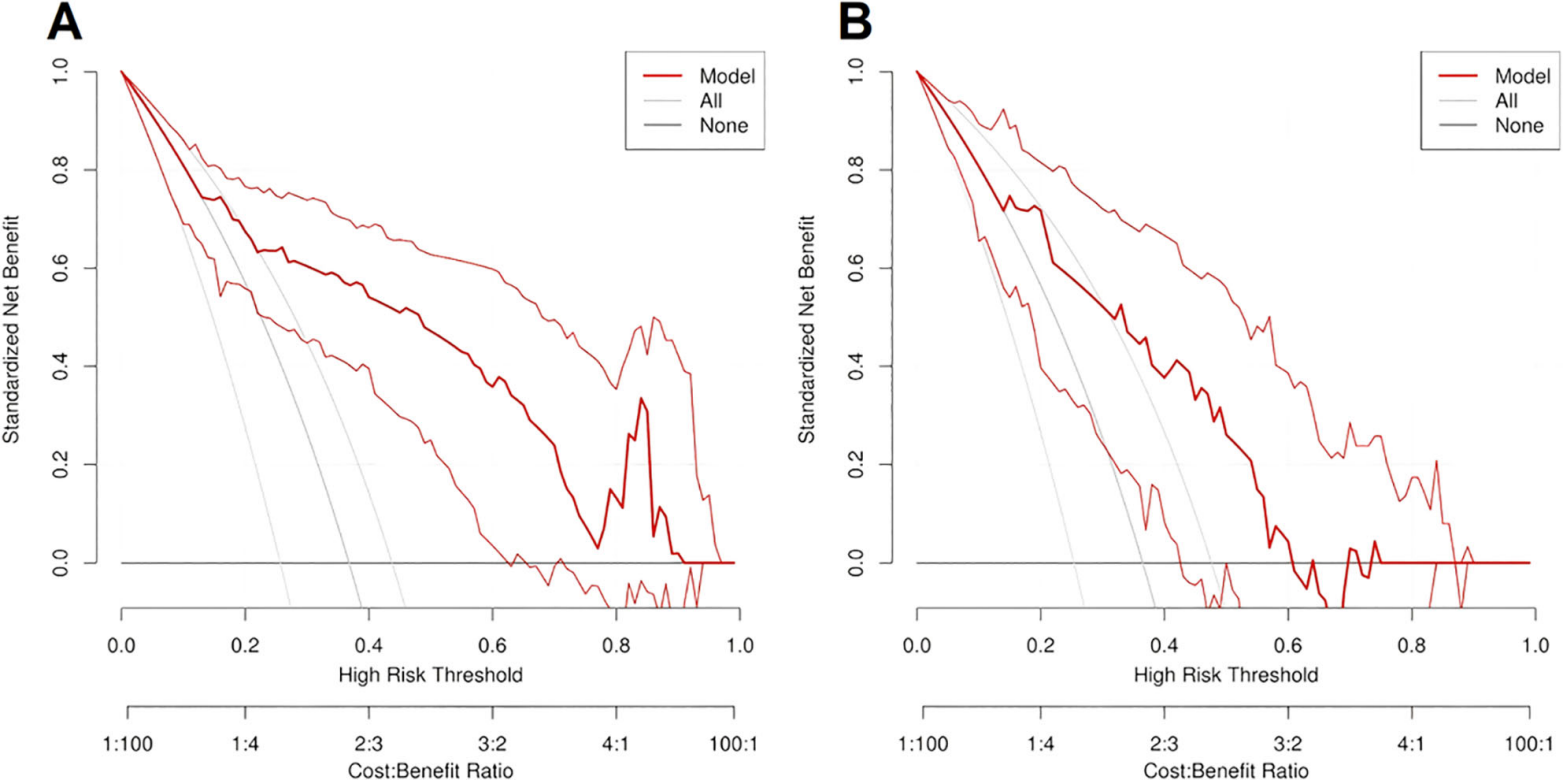
The decision curves of logistic regression model for predicting luminal and non-luminal breast cancer in training set **(A)** and validation set **(B)**.

The ROC curve and nomogram of the logistic regression model in the training and validation sets were shown in **Figure 7**. According to the results shown in **Figure 7**, if the total point is less than 210 points, the patient is categorized as low risk patient (luminal subtype); whereas if the total point is greater than 210 points, the patient is categorized as high risk patient (non-luminal subtype). The decision curves of logistic regression model for discriminating luminal and non-luminal breast cancer in training and validation sets were demonstrated in **Figure 8**. When the threshold was set in the range of 0.13 to 0.61, the net benefit curve was above the reference line, indicating a positive effect of the model on clinical decision making.

## 4 Discussion

In this retrospective study, we validated the application value of DCE-MRI radiomics and heterogeneity analysis in the differentiation of molecular subtypes of luminal and non-luminal breast cancer. Five machine learning models were used to process the data during the model training stage. The logistic regression model demonstrated the best predictive performance among various machine learning models. It showed the capability in distinguishing luminal and non-luminal subtypes with satisfactory reproducibility and reliability.

Different molecular subtypes of breast cancer demonstrate different biological behaviours, including significant differences in pathological histotype, immunophenotype, treatment response, lymph node metastasis and prognosis (14, 15). Recent studies have found that ER and PR status are important markers for determination of breast cancer subtypes, and they are of great value in the evaluation of the response to endocrine therapy and prognosis of breast tumors (16). According to the expression of ER and PR, breast cancer can be divided into the luminal and non-luminal subtypes. The luminal subtype breast cancer is with lower malignancy and recurrence risks, and it is more likely to benefit from endocrine therapy and targeted therapy. Generally, patients with luminal subtype breast cancer have a good prognosis (17, 18).

The non-luminal subtype breast cancer demonstrates worse prognosis and are with a tendency for axillary lymph node metastasis. It has a higher malignancy and recurrence rate than the luminal subtype. Also, the non-luminal subtype breast cancer is relatively insensitive to endocrine therapy or targeted therapy, and it is generally associated with poor prognosis (19–22). Zuo et al. followed 4531 breast cancer patients in 4 hospitals in Beijing and found that the 5-year survival rates of non-luminal subtype breast cancer patients were significantly lower than those of luminal patients (23). Therefore, accurately and preoperatively determination of the molecular subtype of breast cancer is critical to the precise and effective treatment planning and prognosis management.

At present, the determination of the subtypes of breast cancer mainly relies on the IHC measurement of biopsy specimens. However, the conventional biopsy method is invasive, time-consuming and there is a risk of causing subsequent inflammation. Moreover, due to the heterogeneity within the tumor, in the biopsy process only a small portion of lesion area is sampled and examined. Thus it may not be able represent the whole lesion and may bring error and uncertainty (24–26). The ultrasound and breast X-ray photography are common imaging methods for preoperative evaluation of breast cancer. However, for ultrasound examination method, the specificity is low, and it mainly relies on operator’s experience (27). As a consequence, there are high false positive rates in ultrasound breast imaging. For breast X-ray photography, there is low sensitivity for dense breast patients. As a result, these two examination methods are limited in clinical applications. With the development of radiomics and artificial intelligence (AI) in the medical field, the MRI plays a more and more important role in the diagnosis and treatment of breast cancer. The MRI radiomics can provide histopathological information non-invasively with the help of radiomics. The radiomics method can also eliminate the influence of observers’ subjective interpretations of diagnostic results (28).

The DCE-MRI is an effective method in diagnosis of breast cancer by assessing tumor morphology and hemodynamics, which can provide images with high temporal resolution, high spatial resolution and signal-to-noise (SNR) ratio. Recently, the study of radiomic prediction model based on breast DCE-MRI in determination of molecular subtypes of breast cancer is becoming more and more popular (29). Sheng et al. studied 190 Chinese women with invasive ductal breast cancer, the results showed that the combination of radiomics characteristics based on DCE-MRI and clinical data was able to predict molecular subtypes of invasive ductal breast cancer (30). Song et al. studied a machine learning-based prediction model for 300 breast cancer patients (31). The results showed good diagnostic performance in the prediction of the Ki-67 index and histological grade of luminal breast cancer.

Previous study has shown that tumor heterogeneity, including temporal heterogeneity and spatial heterogeneity differs in different subtypes of breast cancer (32). The heterogeneity may be the result of tumor proliferation and interactions of tumor microenvironment factors. It is also suggested that the heterogeneity of breast cancer exists at the level of genomics, proteomics and morphology, finally leading to the different tumor microenvironment (33). The development of a non-invasive tool based on DCE-MRI radiomics and heterogeneous information for the prediction of molecular subtypes of breast cancer is of clinical importance for the next-step accurate and individualized treatment plan.

Five ML models were used in this study to process the training data between luminal and non-luminal subtype breast cancer and were further validated. The best performance was achieved by the logistic regression model with the highest AUC, accuracy, sensitivity and specificity in discriminating luminal and non-luminal subtype breast cancer in the training and validation set. For logistic regression model, the core is to combine a linear model with a logistic function. The logistic function is depicted in Equation 3. The linear output is transformed into a probability value (0-1), and then the classification is made based on this probability.


(3) $$f\left(x\right)=\frac{1}{1+e^{-x}}$$

Logistic regression model has several advantages, including the simple structure, easy to interpret, easy to implement and good robustness. Specifically, when there is a linear relationship between the input features and the target variables, the logistic regression model is able to capture the linear relationship well and achieve higher predictive performance. It learns a linear relationship from the given dataset and performs well when the dataset is linearly separable.

In this study, 8 continuous variable features which are the 1) Heterogeneity, 2) Correlation_angle90_offset1, 3) GLCMEntropy_AllDirection_offset4, 4) Washout, 5) HC_angle0_offset1, 6) HC_AllD_offset7_SD, 7) CS_ angle45_offset4 and 8) CS_AllD_offset1_SD were screened and retained for constructing the machine learning model. The results of point-biserial correlation analysis (PBCA) between the 8 features and the target variables were -0.77, 0.55, 0.49, -0.46, -0.71, 0.32, -0.51 and -0.47, respectively. The results of *t*-test were 0.019, 0.037, 0.032, 0.098, 0.023, 0.151, 0.015 and 0.036, respectively. The results showed that there are linear relationships with significance between the features and the target variables. The logistic regression model based on linear regression analysis is more suitable for dealing with data of linear type. For other machine learning models, they need to introduce more parameters that make the model too complex when dealing with data with a linear relationship. Generally, it results in overfitting or low computational efficiency.

Therefore, the logistic regression model is particularly suited for datasets where there is a linear relationship between variables and features. Thus, in this study, the logistic regression model demonstrated the best prediction performance. The precision, recall and F1 score of each machine learning model were also calculated and shown in **Table 3**. In the validation set, the logistic regression model demonstrated higher precision, recall and F1 score. It further proves the applicability and superiority of the logistic regression model with the dataset of this study.

In the field of machine learning, evaluating model performance based on the validation set is to avoid overfitting. By assessing the model on the validation set, we can more accurately understand the model’s generalization ability on new data. At the same time, using the validation set to evaluate model performance ensures the robustness of the model. The validation set can reveal the stability of the model across different datasets. A model that performs well only on the training set may fail when encountering slightly different data. With the validation set, we can evaluate the model’s adaptability to different data distributions.

There are a number of reasons for the different prediction performances using various ML models, including the differences in data characteristics, model complexity and different parameter settings. Different datasets exhibit various distributions, correlations, noise levels and other characteristics. Different ML models demonstrate distinct processing capabilities to handle the data. The ML model complexity needs to match the complexity of the data. If the model is too simple, it may not capture the complexity of the data, while an over-complex model may lead to overfitting. In addition, for most ML models, some parameters need to be tuned and optimized. Adjustment of parameters in ML modes can significantly impact performance outcomes. The variation in diagnostic efficacy among the ML models in this study may be attributed to the combined influence of these aforementioned factors. In this study, calculated radiomics data may have a linear characteristic, which makes the logical regression model demonstrating better training effect. The reason why logistic regression models often perform better is typically due to their concise linear form, which can capture the core relationships in the data. Techniques such as regularization can effectively prevent overfitting, allowing them to maintain high predictive accuracy while also retaining good generalization capabilities when dealing with complex data. Moreover, the results of the models are easy to understand and interpret. Through the pre-processing step of normalization, overfitting can be prevented. Also, the generalization of the model is improved to further obtain better validation results.

Nomograms visualize the process of working with the regression model as a point scale to predict the occurrence probability of an event. The nomogram maps the scoring of each variable in the model at different values, as well as an overall point scale. Using the nomogram, the clinician can conveniently integrate the points of each variable in the model to obtain a total point, and then predict the probability of an event occurring based on the total point. In this study, the corresponding points can be found in the column-line diagrams in nomogram based on the patient’s histology and heterogeneity characteristics. Then the total point can be calculated and thus the patient’s subtypes of luminal and non-luminal breast cancer can be predicted and differentiated. In clinical applications, patients’ total points will be calculated according to the points of individual radiomics related factors as well as the heterogeneity analysis results. In this way the application of nomogram help the clinician and doctors to predict the molecular subtypes of a breast cancer patient fast, directly and accurately.

For decision curve analysis (DCA), the clinical significance is that it can transform a complex statistical model into an intuitive visual tool, which helps clinicians to understand and apply the model results more easily. Thus the DCA can help clinicians make more accurate prognostic predictions and guide treatment decisions. The decision curve is used to evaluate the performance of the predictive model at different thresholds. It judges the value of the model in clinical decisions by comparing the net gains. The performance of the model was evaluated by comparing the net benefit of the model at different thresholds. Based on the results of the decision curve, the doctor can assess the net benefits of a certain treatment strategy at different thresholds to make a more accurate treatment decision. In this study, when the threshold was set in the range of 0.13 to 0.61, the net benefit curve was above the reference line, indicating a positive effect and clinical benefit of the model on clinical decision making.

In this study, the differences in age, menopausal status, and lymph node metastasis among breast cancer patients of different molecular subtypes are not statistically significant. This is consistent with the findings of Li Wei et al (34). In the heterogeneity analysis, tumor heterogeneity indices and type III curve proportion in non-luminal subtype group were significantly higher than those parameters of luminal group. In non-luminal patients, the tumor is highly malignant with poor differentiation. There may be necrosis inside the lesion, thus the tumor heterogeneity values are higher. Also, there may be neovascularization in highly heterogeneous tumor, which may induce high perfusion and early-stage enhancement during the injection of contrast agent. It may be the main reason of the significantly higher type III curve proportion in non-luminal breast cancer patients. For comparison, for luminal subtype breast cancer patients, the tumor malignancy and heterogeneity index are lower. Thus the plateau patterned type II curve proportion is relatively higher than that of non-luminal patients due to comparatively less neovascularization and less malignant degree. The heterogeneity analysis together with radiomics could provide valuable information in differentiating non-luminal and luminal breast cancer patients.

This study had several limitations. Firstly, the ROI of breast lesions were manually drawn by radiologists, thus the delineation of lesion boundaries may be greatly influenced by individual experience. Secondly, the number of recruited patients with luminal and non-luminal breast cancer is limited. Most of the recruited patients have undergone ultrasound and breast X-ray mammography examinations prior to MRI scans, but a small number of patients are not done in our hospital. We plan to enroll more breast cancer patients and make sure all patients have undergone standard initial ultrasound and breast X-ray examinations. Thirdly, this study did not conduct further analysis of subdivided molecular subtypes of breast cancer, such as luminal A, luminal B, HER2 and triple negative (TN) type. Future research will include more molecular subtypes of breast cancer and further explore the predictive value of combined radiomics features and heterogeneity analysis.

In conclusion, radiomics and heterogeneity analysis based on DCE-MRI have good application potential and value in the prediction of luminal and non-luminal subtypes of breast cancer, with the logistic regression ML model demonstrating the best prediction performance.

## Data Availability

The raw data supporting the conclusions of this article will be made available by the authors, without undue reservation.
